# Therapeutic value of oncology products with a conditional approval from Health Canada: a cross-sectional study

**DOI:** 10.1177/20542704251325314

**Published:** 2025-03-19

**Authors:** Joel Lexchin

**Affiliations:** 1 School of Health Policy and Management, 7991 York University, Toronto, ON, Canada; 2 Faculty of Medicine, University of Toronto, Toronto, ON, Canada

**Keywords:** conditional approval, first-in-class, Health Canada, oncology drugs, orphan drugs, therapeutic value

## Abstract

**Objectives:**

Investigate the additional therapeutic value compared to existing medicines of new oncology drugs given a conditional approval (Notice of Compliance with conditions, NOC/c) by Health Canada using therapeutic ratings from four independent organisations.

**Design:**

A list of all new oncology drugs with an NOC/c from the start of the programme in 1998 to the end of 2023 was constructed. First-in-class and orphan drug status was determined for all drugs. Therapeutic ratings were obtained from the Canadian Patented Medicine Prices Review Board, the French drug bulletin Prescrire International, the French agency Haute Autorité de Santé and the German Institute for Quality and Efficiency in Health Care. If more than one organisation rated the drug, the highest rating was used.

**Setting:**

Canada.

**Participants:**

Oncology drugs with a conditional approval.

**Main outcome measures:**

Additional therapeutic gain compared to existing products.

**Results:**

Fifty-four oncology drugs were approved. Conditions were fulfilled for 29, fulfilment was still pending for 22 and 3 drugs were either discontinued by the manufacturer or placed on restricted access. Eighteen drugs had both orphan drug and first-in-class status. Therapeutic evaluations were available for 50 drugs, and the distribution of additional therapeutic value was examined for the entire group of 50 drugs, for 29 drugs that had fulfilled their conditions and for 18 drugs with both orphan drug and first-in-class status. In the three groups, 8.0%, 10.3% and 11.7%, respectively, offered major therapeutic improvement.

**Conclusions:**

Few new oncology drugs approved through the NOC/c pathway offer major therapeutic improvements over existing drugs.

In an effort to ensure that promising therapies for serious, life-threatening or debilitating illnesses reach Canadians in a timely manner, in 1998 Health Canada developed its Notice of Compliance with conditions (NOC/c) policy. Under this policy, it gives a conditional approval to new drugs (new molecules that have never been marketed in Canada before) or to new indications for already existing drugs. In return, companies commit to additional studies to confirm the benefits of their products.^1^ Whereas from 1998 to 2001, 11% of NOC/c were for oncology products, from 2018 to 2021 that percentage had risen to 94%.^2^ Patented oncology products are the top-selling therapeutic class in Canada, and the mean 28-day cost for treatment with these drugs was over $14,000 in 2022.^3^

It is not clear how therapeutically valuable new oncology drugs approved through the NOC/c pathway are. The conclusion by Martin et al. was that the ‘policy has been successful in providing expedited access to promising new therapies’.^2^ However, only half of all cancer drugs approved by Health Canada that received a positive funding recommendation from the pan-Canadian Oncology Drug Review, the arm of Canada's Drug Agency that conducts health technology assessments for oncology drugs, had evidence of improved overall survival and the survival gains were usually modest.^4^

This retrospective cross-sectional study looks at evaluations from four independent organisations to determine the additional therapeutic value of new oncology drugs approved through the NOC/c pathway.

## Methods

### List of oncology products approved through the NOC/c pathway

A list of all oncology drugs approved through the NOC/c pathway from the time that the policy was developed in 1998 until the end of 2023 was created by examining the NOC/c database maintained by Health Canada.^5^ The brand and generic names of each product, along with the date when they received their NOC/c and the date when the conditions were fulfilled were recorded on an Excel spreadsheet. If the conditions had not been fulfilled at the time the data were gathered, then the data were right censored as of 10 December 2024. Only information about new drugs was recorded, not new indications for existing drugs. This decision was made because therapeutic evaluations are most commonly available for new drugs.

### First-in-class and orphan drug status

Information was gathered about whether the drugs were for rare diseases (orphan drugs) and were first-in-class on the grounds that these features of drugs are often viewed as predictors of significant therapeutic advances. Health Canada does not identify first-in-class status and orphan status for the drugs that it approves. Therefore, Food and Drug Administration (FDA) data were used for both. First-in-class status was identified from a combination of papers by Lanthier et al. and Brown and Wobst and annual FDA reports.^6–9^ Orphan drug status was obtained from a different set of FDA documents.^10,11^

### Therapeutic value

Additional therapeutic value compared to existing products was determined by searching the databases of four organisations that evaluate the efficacy and safety of new drugs: annual reports of the Patented Medicine Prices Review Board, the Canadian agency that evaluates whether the price of patented drugs is excessive; Prescrire International, an independent French drug bulletin; the Haute Autorité de Santé, the French body that assesses the efficacy and safety of drugs; and the Institute for Quality and Efficiency in Health Care, the German health technology assessment organisation (Table 1).^12–15^ All four organisations report in English and use ordinal ratings that allow discrete categorisation of the additional therapeutic value. Their assessments are rigorous and systematic, as is shown by the use of their ratings by other researchers.^16,17^ Evaluations of medicines from the UK National Institute for Health and Care Excellence and the European Medicines Agency were not used because they do not use ordinal ratings in assessing therapeutic gain from new drugs compared to existing ones. Ratings were categorised as major, moderate and minor. If more than one organisation assessed a drug, the highest rating was used.

**Table 1.** Organisations’ rating of therapeutic value.

Organisation	New therapeutic value			Website for definition of therapeutic value
	Major	Moderate	Minor	
Patented Medicine Prices Review Board	Breakthrough, substantial improvement	Moderate improvement – primary and moderate improvement – secondary	Slight or no improvement	http://www.pmprb-cepmb.gc.ca/view.asp?ccid=492#1635
Institut für Qualität und Wirtschaftlichkeit im Gesundheitswesen	Major added benefit, substantial	Considerable added benefit	Minor (more than marginal) added benefit, added benefit not proven, no added benefit, less benefit (than the appropriate comparator therapy)	https://www.iqwig.de/en/about-us/methods/
Prescrire International	Bravo, a real advance	Offers an advantage	Possibly helpful, nothing new, not acceptable	http://english.prescrire.org/en/81/168/46800/0/NewsDetails.aspx
Haute Autorité de Santé	Major, important	Moderate	Minor, nonexistant	https://www.has-sante.fr/upload/docs/application/pdf/2015-02/methodes_has_exigences_communes.pdf

### Data analysis

Descriptive data are reported, and a chi-square test was used to compare the distribution of levels of therapeutic improvement.

All data were collected by a single individual from 10 December 2024 to 10 January 2025 and are available in the Supplemental File.

### Patient involvement and ethics

No patients were involved in this study. Ethical approval was not sought as all data were publicly available.

## Results

Fifty-four new drugs were approved for oncology indications. Conditions were fulfilled for 29, fulfilment was still pending for 22 and 3 drugs were either discontinued by the manufacturer or placed on restricted access. Eighteen drugs had both orphan drug and first-in-class status, 22 only orphan drug status, 5 only first-in-class status and 6 were neither orphan drugs nor first-in-class. There were also three orphan drugs where first-in-class status could not be determined (Table 2).

**Table 2.** Orphan and first-in-class status of oncology drugs^a^.

		First-in-class drug	
		Yes	No
Orphan drug	Yes	18	22
	No	5	6

a No first-in-class status available for three orphan drugs.

Therapeutic evaluations were available for 50 of the 54 drugs – 10 drugs were evaluated by all 4 organisations, 23 drugs by 3 organisations, 9 drugs by 2 organisations and 8 drugs by 1 organisation. The distribution of additional therapeutic value was examined for the entire group of 54 drugs, for the 29 drugs that had fulfilled their conditions and for the 18 drugs with both orphan drug and first-in-class status. In the three groups 8.0%, 10.3% and 11.7%, respectively, offered major therapeutic improvement. In all three groups, the large majority of drugs were rated as having minor additional therapeutic value, and there was no difference in the distribution of therapeutic value between the three groups, *p* = 0.9398, chi-square test (Table 3).

**Table 3.** Distribution of additional therapeutic value.

	Level of additional therapeutic value^a^			No therapeutic gain evaluation
	Minor	Moderate	Major	
Entire group of 54 drugs	40	6	4	4
29 drugs with conditions fulfilled	21	5	3	0
18 drugs with orphan drug and first-in-class status	13	2	2	1

a No different in distribution of therapeutic value regardless of grouping of drugs, *p* = 0.9398, chi-square test.

## Discussion

Regardless of how the data are analysed, most oncology drugs approved through the NOC/c pathway were rated as offering minor additional therapeutic value. This finding does not mean that these drugs have no value, but rather that they are generally no better than existing products. They may offer physicians and patients additional choice or can be used if first-line therapies are unsuccessful or have significant side effects. However, since most of the pivotal trials used to approve these drugs are uncontrolled,^2^ when they are marketed, clinicians have little basis for evaluating how they compare to existing drugs.

The results of this study do not necessarily contradict those of Martin et al. that the NOC/c policy has been successful in providing expedited access to promising new therapies,^2^ since based on the design of premarket trials,^18^ it is unlikely that confirmatory studies would have used a superiority design. Therefore, even if drugs fulfilled their conditions, they might not have been superior to already marketed products.

This study is broadly in agreement with one that found that only 19% of confirmatory studies for 91 cancer drug indications approved by the FDA through its accelerated approval pathway, the equivalent to the NOC/c pathway, demonstrated overall improvement in patient survival.^19^ A second study of 129 oncology drug indications with accelerated approval concluded that most did not demonstrate survival benefits within 5 years of approval.^20^ Looking at all drugs approved through the FDA's accelerated approval pathway or the European Medicines Agency's equivalent between 1 January 2007 and 31 December 2021, only 38.9% and 37.5%, respectively, demonstrated high therapeutic value.^21^ These percentages are at the high end of what studies looking at expedited approvals in Canada, Europe, France, Germany and the United States have found.^22^

The conclusion that few oncology drugs approved through the NOC/c pathway offer substantial therapeutic gain may be a reflection of the ambiguity of the standards that admit drugs into this pathway. The analysis by McPhail et al. of oncology drugs with conditional approval concluded that eligibility criteria are not clearly defined and are inconsistently applied, undermining the justification of earlier market access based on incomplete evidence.^23^ If drugs are being inappropriately deemed eligible for conditional approval, it is not surprising that they do not demonstrate any significant new benefit.

### Limitations

The data for this study were gathered and interpreted by a single person, which may have introduced inadvertent transcription and interpretation errors. Results for new oncology indications for existing drugs may differ from those for new drugs. The results of this study do not necessarily apply to other classes of drugs receiving an NOC/c, although at present the pathway is almost exclusively used for oncology drugs.

## Conclusion

Few new oncology drugs approved through the NOC/c pathway offer major therapeutic improvements over existing drugs. This finding reinforces the question about the criteria used for entry into this pathway and how well the money used to pay for many of these drugs is being spent.

## Declarations

## Supplemental Material

sj-pdf-1-shr-10.1177_20542704251325314 - Supplemental material for Therapeutic value of oncology products with a conditional approval from Health Canada: a cross-sectional study Supplemental material, sj-pdf-1-shr-10.1177_20542704251325314 for Therapeutic value of oncology products with a conditional approval from Health Canada: a cross-sectional study by Joel Lexchin in JRSM Open

sj-docx-2-shr-10.1177_20542704251325314 - Supplemental material for Therapeutic value of oncology products with a conditional approval from Health Canada: a cross-sectional study Supplemental material, sj-docx-2-shr-10.1177_20542704251325314 for Therapeutic value of oncology products with a conditional approval from Health Canada: a cross-sectional study by Joel Lexchin in JRSM Open
