# Supplementary material for: Therapeutic value of oncology products with a conditional approval from Health Canada: a cross-sectional study
Source: JRSM Open. 2025 Mar 19;16(3):20542704251325314. doi: 10.1177/20542704251325314 (PMC11924082; doi:10.1177/20542704251325314)
Supplement: sj-pdf-1-shr-10.1177_20542704251325314 - Supplemental material for Therapeutic value of oncology products with a conditional approval from Health Canada: a cross-sectional study [file sj-pdf-1-shr-10.1177_20542704251325314.pdf]

Generic name	Date NOC/c	Date NOC/c fulfilled	Date NOC/c suspended or indication removed	Reason for suspension, etc.	Significant therapeutic advance (STA)/moderate therapeutic avance (MTA)/no therapeutic advance (NTA)	Number of therapeutic evaluations	Orphan (no=0, yes=1)	First in class (no=0, yes=1)
alectinib	2016-09-28	2018-09-26			MTA	3	1	0
amivantamab	2022-03-30	2024-12-10			NTA	3	0	1
avelumab	2017-12-18	2021-01-13			NTA	3	1	0
blinatumomab	2015-12-22	2017-11-16			NTA	3	1	1
bortezomib	2005-01-27	2007-09-11			NTA	3	0	1
bosutinib	2014-03-07	2017-08-02			NTA	3	1	0
brentuximab vedotin	2013-02-01	2020-05-12			NTA	3	1	1
brigatinib	2018-07-26	2022-07-20			NTA	3	1	0
capmatinib	2022-05-26	2024-12-10			NTA	1	1	0
cemiplimab	2019-04-10	2023-08-15			NTA	3	0	0
ceritinib	2015-03-27	2024-12-10			NTA	4	1	1
ciltacabtagene autoleucel	2023-02-09	2024-12-10			NTA	1	1	
crizotinib	2012-04-25	2015-11-18			NTA	3	1	1
daratumumab	2016-06-29	2018-10-10			NTA	4	1	1
dasatinib	2007-03-26	2009-11-19			STA	3	1	0
dostarlimab	2021-12-23	2024-07-23			NTA	1	0	0
durvalumab	2017-11-03	2024-12-10			NTA	4	0	0
elranatamb	2023-12-06	2024-12-10			NTA	1	1	0
enasidenib	2019-02-06		2023-06-30	Discontinued by company		0	1	1
entrectinib	2020-02-10	2024-12-10			NTA	2	1	0
epcoritamab	2023-10-13	2024-12-10			NTA	1	1	0
erdafitinib	2019-10-25	2024-09-24			NTA	1	0	1
gefitinib	2003-12-17	2009-12-18			NTA	3	0	1
glofitamab	2023-03-24	2024-12-10				0	0	0
idecabtagene [illegible]	2021-05-26	2024-12-10			NTA	2	1	1
idelalIsib	2015-03-27	2020-04-21			NTA	3	1	1
imatinib	2001-09-20	2017-11-24			STA	3	1	1
infigratinib	2021-09-27	2024-12-10				0	1	0
larotrectinib	2019-07-10	2024-12-10			STA	3	1	1
lorlatinib	2019-02-22	2022-05-20			MTA	4	1	0
lurbinectedin	2021-09-29	2024-12-10				0	1	0
nelarabine	2007-09-22	2020-01-22			STA	1	1	
nilotinib	2008-09-09	2011-11-30			NTA	2	1	0
ofatumumab	2012-03-09	2024-12-10			NTA	3	1	0
olaparib	2016-04-29	2018-05-02			MTA	4	1	1

olaratumab	2017-11-23		2020-10-08	Discontinued by company	MTA	2	1	0
osimertinib	2016-07-05	2018-01-19			MTA	4	1	0
palbociclib	2016-03-16	2017-11-17			NTA	4	0	1
panitumumab	2008-04-03	2015-02-19			NTA	3	0	0
pembrolizumab	2015-05-19	2017-12-22			NTA	3	1	1
pemigatinib	2021-09-17	2024-12-10			NTA	2	1	0
polatuzumab vedotin	2020-07-09	2023-01-20			NTA	3	1	1
ponatinib	2015-04-02	2022-10-03			MTA	3	1	1
pralatrexate	2018-10-26	2024-12-10			NTA	1	1	0
pralsetinib	2021-06-30	2024-12-10			NTA	3	1	0
romidepsin	2013-10-16		2023-03-04	Placed under a Restricted Access Program as of March 14, 2023 because the confirmatory study did not support use	NTA	2	1	0
selpercatinib	2021-06-15	2024-12-10			NTA	2	1	0
sorafenib	2006-07-28	2009-06-12			NTA	3	1	
sotorasib	2021-09-10	2024-12-10			NTA	3	1	1
tafasitamab	2021-08-19	2024-12-10			NTA	4	1	1
teclistamab	2023-07-26	2024-12-10			NTA	2	1	1
tepotinib	2021-05-27	2024-12-10			NTA	2	1	0
trastuzumab deruxtecan	2021-04-15	2024-07-09			NTA	4	0	0
venetoclax	2016-09-30	2020-01-13			NTA	4	1	1
