# Supplementary material for: Therapeutic value of oncology products with a conditional approval from Health Canada: a cross-sectional study
Source: JRSM Open. 2025 Mar 19;16(3):20542704251325314. doi: 10.1177/20542704251325314 (PMC11924082; doi:10.1177/20542704251325314)
Supplement: sj-docx-2-shr-10.1177_20542704251325314 - Supplemental material for Therapeutic value of oncology products with a conditional approval from Health Canada: a cross-sectional study [file sj-docx-2-shr-10.1177_20542704251325314.docx]

**Supplemental File**

**Title:** Supplemental File for Therapeutic value of oncology products with a conditional approval from Health Canada: a cross-sectional study

**Description:** Supplemental material – all data collected – for Therapeutic value of oncology products with a conditional approval from Health Canada: a cross-sectional study in Journal of the Royal Society of Medicine Open.
